# Role of aldo‐keto reductase family 1 member B1 (AKR1B1) in the cancer process and its therapeutic potential

**DOI:** 10.1111/jcmm.15581

**Published:** 2020-07-06

**Authors:** Reza Khayami, Seyyed Reza Hashemi, Mohammad Amin Kerachian

**Affiliations:** ^1^ Medical Genetics Research Center Mashhad University of Medical Sciences Mashhad Iran; ^2^ Department of Medical Genetics Faculty of Medicine Mashhad University of Medical Sciences Mashhad Iran; ^3^ Student Research Committee Mashhad University of Medical Sciences Mashhad Iran; ^4^ Cancer Genetics Research Unit Reza Radiotherapy and Oncology Center Mashhad Iran

**Keywords:** AKR1B1, aldose reductase, biomarker, cancer, methylation, tumour

## Abstract

The role of aldo‐keto reductase family 1 member B1 (AKR1B1) in cancer is not totally clear but growing evidence is suggesting to have a great impact on cancer progression. AKR1B1 could participate in a complicated network of signalling pathways, proteins and miRNAs such as mir‐21 mediating mechanisms like inflammatory responses, cell cycle, epithelial to mesenchymal transition, cell survival and apoptosis. *AKR1B1* has been shown to be mostly overexpressed in cancer. This overexpression has been associated with inflammatory mediators including nuclear factor kappa‐light‐chain‐enhancer of activated B cells (NFκB), cell cycle mediators such as cyclins and cyclin‐dependent kinases (CDKs), survival proteins and pathways like mammalian target of rapamycin (mTOR) and protein kinase B (PKB) or AKT, and other regulatory factors in response to reactive oxygen species (ROS) and prostaglandin synthesis. In addition, inhibition of *AKR1B1* has been shown to mostly have anti‐cancer effects. Several studies have also suggested that *AKR1B1* inhibition as an adjuvant therapy could render tumour cells more sensitive to anti‐cancer therapy or alleviate the adverse effects of therapy. *AKR1B1* could also be considered as a potential cancer diagnostic biomarker since its promoter has shown high levels of methylation. Although pre‐clinical investigations on the role of *AKR1B1* in cancer and the application of its inhibitors have shown promising results, the lack of clinical studies on *AKR1B1* inhibitors has hampered the use of these drugs to treat cancer. Thus, there is a need to conduct more clinical studies on the application of *AKR1B1* inhibitors as adjuvant therapy on different cancers.

## 1 INTRODUCTION

**Figure 1:**
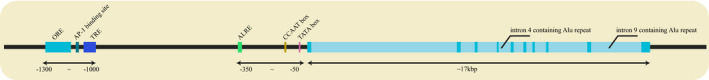
The structure of AKR1B1 gene

Aldo‐keto reductase family 1 (AKR1) is a family of aldose keto reductase (AKR) superfamily consist of 16 families which are divided into subfamilies and members based on their amino acid sequence identity. Fifteen members of aldose reductases have been recognized in humans to date.^1^, ^2^, ^3^ AKR1 subfamily B is comprised of three members: AKR1B1 (AR; ADR; ALR2; ALDR1; HGNC: 381; EC: 1.1.1.21), AKR1B10 and AKR1B15.^4^, ^5^ The *AKR1B1* gene located on 7q33 is 18 kb long and its coding transcript contains 10 exons.^6^ Its mRNA transcript is 1,384 nucleotides long and codes a 316‐amino acid protein.^6^ In addition to a TATA (TATTTA) box at −37 and a CCAAT box at −104 in the promoter, the *AKR1B1* gene contains two Alu repeats in intron 1 and two Alu repeats in intron 4 and 9, respectively.^7^ An androgen‐like response element is also located at 396 to 382 nucleotides upstream of the gene.^6^, ^8^ Three osmotic response elements (OreA, OreB and OreC) are found at approximately 1 kb upstream of the transcription start site in a 132 bp region.^9^ An activator protein 1 (Ap‐1) binding site is positioned approximately 1100 bp upstream of the gene.^8^ Thyroid receptor element (TRE) is located in the region from 1099 to 1028 upstream of the transcription start site^10^ (Figure 1). *AKR1B1* is translated to a monomeric in a region of 36 kD enzyme, which is located in the cytoplasm. This enzyme consumes reduced nicotinamide adenine dinucleotide phosphate (NADPH) and converts it to nicotinamide adenine dinucleotide phosphate (NADP^+^) in the process of reducing aldehyde compounds to alcohol.^11^ AKR1B1 plays an important role in glucose metabolism and osmoregulation and has a supportive role in the reduction of superoxides and toxic materials.^12^ Because of the diverse roles in body metabolism and especially its association with NFκB, AKR1B1 has been suggested to contribute in tumorigenesis.^13^, ^14^, ^15^

Additionally, AKR1B1 is involved in the polyol pathway. In this pathway in hyperglycaemic condition aldose reductase reduces glucose to sorbitol by consuming NADPH and later sorbitol is converted to fructose by sorbitol dehydrogenase. This was first reported by Hers in 1965.^16^ NADPH is also needed for the conversion of oxidized glutathione (GSSG) to reduced glutathione (GSH) which is an antioxidant. Concretely, some aldose reductase inhibitors have been shown to increase GSH levels.^17^ The excessive sorbitol itself could play a role in osmotic stress and even the phosphorylated fructose could lead to the production of advanced glycation end products (AGEs) which eventually may increase ROS. Consequently, ectopic activation of the polyol pathway could result in different diabetic complications.^18^, ^19^, ^20^ AKR1B1 association with GSH does not end up here. The enzyme could also reduce lipid peroxidation products especially the ones that conjugate with GSH.^21^ For example, by the action of cytokines, growth factors and lipopolysaccharides, lipid peroxidation products could ultimately be synthesized. These compounds could be converted to 4‐hydroxynonenal (HNE). HNE could conjugate with GSH producing 3‐glutathionyl‐4‐hydroxynonanal (GS‐HNE), which could be converted to GS‐dihydroxynonane (GSDHN).^22^ AKR1B1 together with GSDHN may activate phospholipase C/ protein kinase C (PLC‐PKC) pathway, which stimulates NFκB. Hence, lipid aldehydes could affect the NFκB pathway and as a result, AKR1B1 activates the NFκB pathway by reducing GSH‐aldehydes.^23^, ^24^ This may prove a point that AKR1B1 could have a role in cancer promotion through NFκB activation, which has the ability to promote tumorigenicity in several cancers.^25^, ^26^

**Figure 2:**
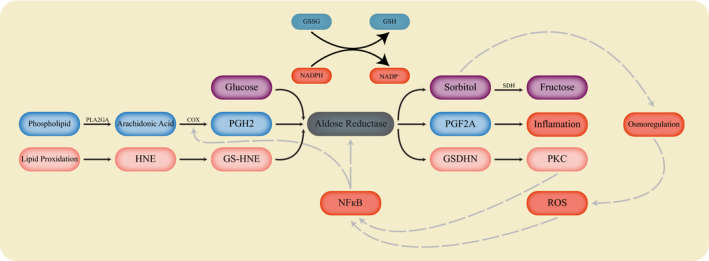
The aldose reductase activity

AKR1B1 is also involved in prostaglandin synthesis. In normal conditions, phospholipid is turned to arachidonic acid in a reaction, catalysed by phospholipases A2 (PLA2G) enzyme. Then, arachidonic acid is converted to prostaglandin H2 (PGH2) by the help of cyclooxygenase 1 (COX1) and cyclooxygenase 2 (COX2). AKR1B1 consumes NADPH and converts PGH2 to prostaglandin F2alpha (PGF2A). Hence, it has been proposed that increased amounts of ROS could lead to the activation of NFκB which acts as a tissue factor (TF) for the expression of COX2. This results in the formation of excessive amounts of PGH2. On the other hand, NFκB could enhance *AKR1B1* expression which causes the production of increased PGF2A from PGH2 by *AKR1B1*. Consequently, excessive amounts of PGF2A would lead to inflammation which could end up with increased tumorigenicity (Figure 2).^27^, ^28^

Although the various roles of AKR1B1 have been identified in different metabolic and physiological processes, such as glucose metabolism, inflammation and prostaglandin synthesis, its true function in cancer still remains unknown. Several studies have been conducted to unveil the role of AKR1B1 in different cancers including colorectal, breast, pancreatic and hepatocellular carcinoma. In this review, we summarized the recent understandings on this topic and the improvements that could be made in cancer treatment by using AKR1B1.

## 2 TUMORIGENESIS OF *AKR1B1* GENE

### 2.1 *AKR1B1* in colorectal cancer

Several studies have been conducted on measuring *AKR1B1* expression to find out more about its role in cancer. It has been demonstrated that *AKR1B1* is expressed universally throughout the body.^29^ There is still debate on how the expression of *AKR1B1* affects cancer but some evidence suggests that expression of *AKR1B1* in colorectal cancer (CRC) could be different depending on the stages, types and invasiveness of tumours, at least in mice models or cell lines. For example, in vivo studies have indicated higher *AKR1B1* levels in invasive tumour cells in mice having colon cancer with *Trp53* deletion in comparison with normal and non‐invasive models.^30^ In colon cancer cell lines, overexpression of *AKR1B1* has been described in the metastatic SW620 cell line compared to non‐metastatic SW480 cells ^29^ and several studies have highlighted a lower expression of *AKR1B1* in SW480 and HT29.^17^, ^31^, ^32^ In another study conducted on HT‐29 and SW480, *AKR1B1* mRNA expression was seen in SW480 without any protein expression; however, no *AKR1B1* mRNA expression was found in HT‐29 while it was seen on protein level.^33^ Interestingly, in colorectal tissues, either no alteration or down‐regulation of AKR1B1 has been reported, for example, by using RT‐PCR, Kropotova *et al* for the first time reported a reduction of AKR1B1 in 10 per cent of tumour samples.^34^ Besides, down‐regulation of AKR1B1 in protein levels has been reported in adenocarcinoma samples.^31^, ^35^ Furthermore, a significantly different expression of *AKR1B1* and *S100P* was found between lymph nodes categorized as Dukes’ stage B groups and controls.^36^ Surprisingly, Nakarai *et al* reported that no differential expression of *AKR1B1* was observed between inflammatory, tumour and non‐tumour tissues in mRNA levels.^36^ Another study also showed the same results by microarray analysis.^29^ Despite the fact that there is still no clear correlation between the expression of *AKR1B1* and tumour creation in CRC tissues, several evidence suggest that AKR1B1 could play a role in the tumorigenesis of CRC. Accordingly, several mechanisms have also been postulated.

#### 2.1.1 Evidence for the role of *AKR1B1* in inflammation

It has been proposed that ROS creation could result in the activation of inflammatory TFs such as NFκB, resulting in carcinogenesis. In this regard, it has been suggested that *AKR1B1* could have a fundamental role in the regulation of ROS.^37^ Consistently, ROS creation has been shown to be reduced after the knockdown of *AKR1B1* in CRC.^29^ *AKR1B1* has also been found to be involved in the NFκB regulation. Bioinformatics analysis has demonstrated that ‘regulation of cytokine production’ was a significantly enriched Gene Ontology term among the *AKR1B1* overexpressing samples in CRC *AKR1B1* has also been found to be associated with a set of inflammatory‐related genes.^29^ Furthermore, silencing AKR1B1 in CRC cells has been found to cause a reduction in translocation of p65 and p50 NFκB subunits which were partially restored after renovating *AKR1B1* expression. Reduced activity and transcription of NFκB have also been reported after silencing AKR1B1. Inhibition of AKR1B1 in Caco‐2 cells treated with growth factors has resulted in the reduction of NFκB.^29^ Along with this, inhibition of AKR1B1 with Fidarestat resulted in the inhibition of Cox‐2 and iNOS in both ApcMin/+ mice under HFD and C57BL/KsJ‐*db/db* obese mice which contributed to low NFκB levels in cells.^38^, ^39^ NFκB binding protein has also been reported to be reduced in the metastatic liver of mice injected with HT29 or KM20 cells.^40^

Another evidence that suggests AKR1B1 has a role in CRC inflammation is the notion that AKR1B1 plays a role in the synthesis of prostaglandins. In CRC, a study reported that COX2 in Caco‐2 cells is required for the synthesis of prostaglandin E2 (PGE2). Fibroblast growth factor (FGF) and platelet‐derived growth factor (PDGF) could induce PGE2 synthesis in Caco‐2 cells via COX2. This effect has been shown to be abolished by AKR1B1 inhibition. On the other hand, after inhibition of AKR1B1, such an impact was not seen in cells without COX2.^24^ Tumour necrosis factor‐alpha (TNF‐a) has also been elucidated to induce PGE2 and COX2 while AKR1B1 inhibition abrogated the effect in Caco‐2 cells. Besides, AKR1B1 inhibition hindered the PKC and NF‐κB activation induced by TNF‐a.^41^ Taken together, these data suggest that AKR1B1 could have a regulatory role on the inflammatory responses and the carcinogenesis through manipulation of ROS, NFκB and PGE2 synthesis in CRC.

#### 2.1.2 Evidence for the effect of *AKR1B1* in cell cycle

It has been suggested that growth factor‐induced ROS could activate AKT.^42^ This could also result in the overexpression of G1‐S regulatory proteins such as C‐Myc and its downstream targets including E2F‐1, cyclin‐dependent kinase (CDKs) and cyclins.^40^, ^43^ The inhibition of AKR1B1 abrogates these outcomes.^43^ This indicates that AKR1B1 may play a role in the progression of the cell cycle in CRC. These findings have been confirmed in several studies. For example, it has been proposed that AKR1B1 inhibition could arrest the proliferation of Caco‐2 cells at S phase^24^ and the accumulation of cells at G1 phase has been observed in HT‐29, SW480 and HCT‐116 cells.^43^ Cyclins D1 and E, cdk4, proliferating cell nuclear antigen (PCNA), E2F and C‐Myc were also suppressed following AKR1B1 inhibition.^43^ Similarly, in another study, silencing *AKR1B1* slowed down the progression of the cell cycle, reducing tumorigenesis in CRC as the cells transferred from G1 to S with a delay compared to normal cells.^29^ It has also been reported that *AKR1B1* knockdown raised the cyclin E levels in CRC with the cells in the starved state experiencing elevation in cyclin E levels compared to the cells in the released state. The study proposed that the increase in cyclin E was independent of transcriptional up‐regulation as Rb phosphorylation did not change.^29^ This is in contrast to the report published by Ramana *et al* ^43^ who suggested that AKR1B1 inhibition could impede Rb phosphorylation, induced by growth factors. Since AKR1B1 could increase tumorigenesis by inducing cell cycle progression in CRC, its inhibition could be used as a therapeutic approach in the treatment of cancer.

#### 2.1.3 Evidence for the role of *AKR1B1* in mTOR pathway

Multiple lines of evidence suggest that tumour progression could be manipulated by AKR1B1 through modulating a complicated network of miRNAs, proteins and pathways. Hence, AKR1B1 inhibition might be useful in the treatment of cancer. It has been proposed that AKR1B1 inhibition by Fidarestat could prevent tumour growth induced by growth factors in CRC. Epidermal growth factor (EGF) and *basic fibroblast growth factor* (bFGF) could reduce programmed cell death protein 4 (PDCD4) protein, a tumorigenesis suppressor, by inducing the expression of its target miRNA, *mir‐21*, in CRC. These growth factors could also increase ROS through the phosphorylation of PLC. Interestingly, the Inhibition of AKR1B1 has been demonstrated to down‐regulate *mir‐21* and abrogate these effects. Furthermore, PDCD4 could be increased in CRC by AP‐1 down‐regulation, a transcription factor regulating *mir‐21*.^44^ It has been reported that Forkhead box O3A (FOXO3a) expression could inhibit AP‐1 activation and *mir‐21* expression. FOXO3A expression has also been reported to be raised after AKR1B1 inhibition in CRC cell lines.^45^, ^46^

**Figure 3:**
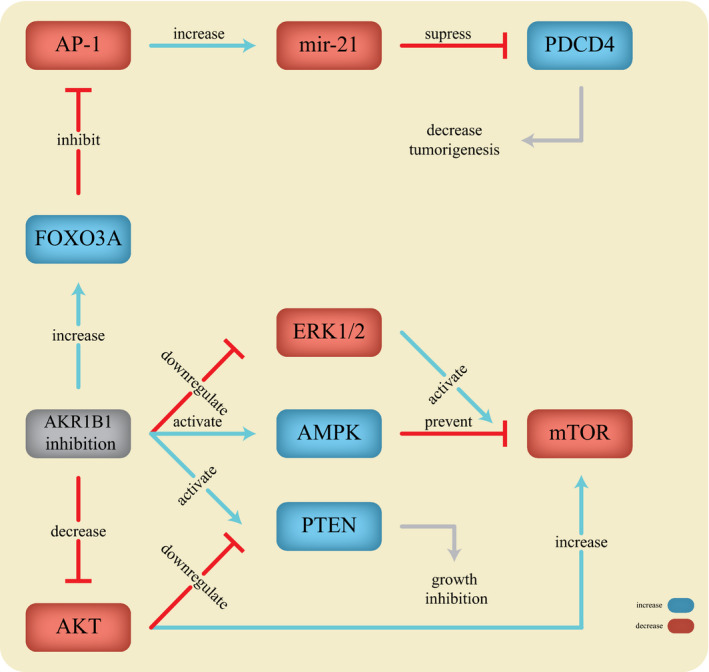
The action of AKR1B1 in tumorigenesis

AKR1B1 inhibition could also prevent tumorigenesis via mTOR inhibition. AKR1B1 inhibition not only could activate phosphatase and tensin homolog (PTEN) through the inhibition of phosphorylation but also could increase its expression. Thus, AKR1B1 inhibition could suppress cell proliferation by the induction of PTEN and FOXO3A which are negative regulators of PI3K/AKT/AP‐1 (Figure 3).^46^ Several other studies have also indicated that AKR1B1 inhibition could reduce the phosphorylation of AKT,^38^, ^43^ and increase PKC B2 in both small and large intestines in Apc^Min/+^ mice under high‐fat diet.^38^ However, it has been shown that AKR1B1 blockage could hinder the PKC activation, triggered by either growth factors or TNF‐a.^24^, ^41^

Alternatively, AKR1B1 inhibition could prevent activation of the mTOR pathway through 5' adenosine monophosphate‐activated protein kinase (AMPK) activation as it prevents phosphorylation of mTOR, Raptor, eIF4E, S6K and 4E‐BP1, thereby inhibiting tumour growth. AKR1B1 inhibition has also been reported to increase P53 protein, a tumour suppressor, which could inhibit mTOR activity.^47^, ^48^

It has been also suggested that ERK stimulation has been proposed to activate the mTOR pathway.^49^ In this regard, it has been shown that silencing *AKR1B1* in HCT‐116 cells resulted in a lower proliferation, migration and wound closure as well as a lower phosphorylation of ERK1/2 in MAP kinase cascade.^29^ This may be due to mTOR deactivation. These data suggest that inhibition of aldose reductase could prevent tumour growth via mTOR inhibition by different mechanisms. This could prove the usefulness of AKR1B1 inhibitors to design new drugs for target therapy in CRC.

#### 2.1.4 Evidence for the role of *AKR1B1* in liver metastasis of CRC

There is evidence suggesting that AKR1B1 inhibition could hinder liver metastasis in CRC. Tammali *et al* reported that AKR1B1 inhibition prevented the migration, invasion and adhesion in HT29 and KM20 colon cells induced by EGF and FGF. Inhibition of AKR1B1 led to down‐regulation of inter‐cellular adhesion molecule‐1 (ICAM‐1), vascular cell adhesion molecule‐1 (VCAM‐1) and vascular endothelial‐cadherin triggered by EGF or FGF. AKR1B1 inhibition has also inhibited liver metastasis in athymic nude mice injected with HT29 or KM20 cells. AKR1B1 inhibition further inhibited MMP2, cyclin D1, CD31, CD34 and NFκB binding protein in metastatic liver.^40^

Altogether, *AKR1B1* could induce malignancy in CRC by affecting cell proliferation, migration and collagen expression. It could also manipulate inflammatory responses by increasing ROS creation and NFκB activation, facilitating tumorigenesis. Moreover, the ability of *AKR1B1* to play a role in cell cycle and cyclin regulation may add to the evidence of its oncogenic properties. Further research is still necessary to uncover more information about aldose reductase and its roles in cancerous colorectal cells.

### 2.2 *AKR1B1* in breast cancer

In 2006, Saraswat *et al* indicated overexpression of AKR1B1 in several cancers such as breast, ovarian, cervical and rectal cancer using immunoblotting.^50^ Similarly, another study reported the up‐regulation of AKR1B1 in triple‐negative breast cancer and the basal subtype of breast cancer cell lines. They found that AKR1B1 was expressed in basal‐like breast cancer (BLBC) at the protein level while it was absent in luminal cell lines.^51^ Moreover, AKR1B1 overall has shown more activity in red blood cells (RBCs) and tissues of breast cancer patients in all three grades of primary surgical and post‐chemotherapy samples.^52^ In contrast, in another study, AKR1B1 was reported to be suppressed in breast cancer tissue in comparison with normal breast tissue.^53^ Although studies measuring *AKR1B1* expression could not clearly highlight its effect on breast cancer, several evidence suggest that AKR1B1 could play a significant role in breast cancer tumorigenesis and epithelial to mesenchymal transition (EMT). For example, results of SNP array analysis in breast cancer patients who consume Betel Quid, a mixture of certain substances, with carcinogenic properties,^54^ were enriched with aldose reductase activity.^55^

Furthermore, expression of *ZEB1*, a master regulator of EMT, in breast cancer cell line MDA‐231 has been shown to positively correlate with *AKR1B1* expression, strengthening the association of *AKR1B1* with EMT.^30^ It has been also indicated that Twist2 is highly expressed in breast cancer and nuclear Twist2 plays a role in inducing EMT in breast cancer.^56^ Moreover, positive correlation and direct interaction of Twist2 and AKR1B1 have been indicated in breast cancer. Knockdown of Twist2 in BLBC cells has been seen to restore E‐cadherin and reduce *AKR1B1* expression suggesting that Twist2 could regulate *AKR1B1* expression as a TF. A negative association has also been observed between E‐cadherin and AKR1B1 expression since suppression of one end up with acceleration of the other one.^51^ Consequently, induced expression of *AKR1B1* by Twist2 could regulate E‐cadherin which its suppression has been seen to induce migration and invasion regardless of EMT in breast cancer.^51^, ^57^ Furthermore, some evidence suggests that AKR1B1 might also play a role in inflammatory responses as its inhibition could be interrupted with inflammation, triggered by chemokines, growth factors and inflammatory cytokines such as TNF alpha.^55^ It has been reported that TNF alpha and interleukin 1 beta could induce Twist2 and RelA expression. In addition, RelA may directly up‐regulate the expression of Twist2 by binding to its promoter, indicating the role of NFκB.^51^ Consequently, it has been shown that suppression of *AKR1B1* inhibits the expression of *RelA* and *Twist2* while its overexpression could induce *RelA* and *Twist2* in various cell lines of breast cancer. Additionally, Wu *et al* have found that Twist2 could directly bind to *AKR1B1* promoter in an E‐box (CANNTG), located at −997. Furthermore, their research has shown that the inhibition of AKR1B1 suppresses PGF2a, an NFκB activator, causing NFκB to decrease.^51^

Taken together, these data show that AKR1B1 could be involved in a positive regulatory feedback mechanism between NFκB and Twist2 contributing to EMT. It has been suggested that EMT could cause tumour cells to obtain cancer stem cell (CSC) properties. Therefore, AKR1B1 associated with the maintenance of CSCs and is required for tumorigenicity and metastasis of breast cancer.^51^

In addition to colorectal and breast cancers, the expression level of *AKR1B1* has been studied in other cancers although some have shown over‐ or low‐ expression.

### 2.3 Other cancers

#### 2.3.1 Overexpression of *AKR1B1*

##### Pancreas cancer

It has been demonstrated that the β2‐adrenergic receptor (B2‐AR) activation by chronic stress could increase pancreas cancer through interactions with AKR1B1.^58^ AKR1B1 alongside B2‐AR has been shown to be expressed more in the cytoplasm of pancreatic cancer cells while a moderate expression of AKR1B1 and B2‐AR in the nucleus and the membrane of cells has been demonstrated. It has been reported that the expression of AKR1B1 could regulate B2‐AR expression in a negative feedback mechanism. A decrease in B2‐AR was observed after overexpression of AKR1B1 while B2‐AR overexpression led to elevated levels of AKR1B1. Consequently, AKR1B1 overexpression was shown to result in inhibition of apoptosis and proliferation induction in pancreatic cancer. Direct interaction of B2‐AR and AKR1B1 has also been seen in BXPC‐3 pancreatic cells as they both were co‐localized in pancreatic tissues and induced tumorigenesis. In this regard, they both increased phosphorylated ERK1/2 levels while inhibition of B2‐AR caused a reduction in AKR1B1 and p‐ERK1/2 expression. Furthermore, AKR1B1 was more expressed in cells being in the S phase in comparison to cells in the G1 phase, supporting the evidence that AKR1B1 could promote proliferation and hinder apoptosis through ERK1/2 pathway.^58^ Moreover, a study by Schwab *et al* have suggested that there is an association between *ZEB1*, a gene encoding a TF and *AKR1B1,* at least in expression levels in pancreatic cancer. Lower levels of AKR1B1 was seen in mice with pancreatic tumours after knockdown of ZEB1 in comparison with controls; however, no direct effect was found between *AKR1B1* and ZEB1 suggesting an indirect interaction.^30^ Interestingly, *AKR1B1* overexpression was associated with decreased survival in patients with pancreatic cancer.^59^ Further research is required to elucidate the exact mechanism underlying the AKR1B1 role in tumour progression in pancreatic cancer.

##### Lung cancer

There is evidence suggesting that AKR1B1 could promote tumour progression in lung cancer. For example, *AKR1B1* up‐regulation has been seen in lung cancer.^60^ In addition, *AKR1B1* expression showed positive correlations with ZEB1 and lymph node involvement in lung cancer cell lines suggesting a role in EMT. Further knockdown of *AKR1B1* in the A549 lung cancer cell line resulted in the reduction of EMT phenotype. Moreover, using immunohistochemistry (IHC), negative correlation was found between AKR1B1 and E‐Cadherin in tissues from resected non‐small cell lung cancer (NSCLC) patients. *AKR1B1* also showed more amounts in airway epithelial cells of smoker subjects in comparison to non‐smoker ones. These data suggested *AKR1B1* expression as a poor prognosis sign in NSCLC.^30^ Furthermore, 12‐O‐tetradecanoylphorbol‐13‐acetate (TPA), a potent tumour promoter, was shown to indirectly increase the expression of AKR1B1 by augmenting the expression of protein kinase C (PKC) and NFκB. Hence, inhibition of AKR1B1 could assist to treat cancer by reducing tumour growth.^61^

##### Hepatocellular carcinoma

In 1995, AKR1B1 up‐regulation was demonstrated in vivo and in vitro in hepatocellular carcinoma (HCC). Besides, the expression level of AKR1B1 increased in 3’‐methyl‐4‐dimethyl‐aminoazobenzene (3‘‐Me‐DAB)‐induced HCC. Inhibition of AKR1B1 caused hepatoma cells to become more sensitive to 3‐deoxyglucosone and glyceraldehyde, suggesting a role for AKR1B1 in cancer resistance in hepatoma cells.^62^ The up‐regulation of AKR1B1 in human HCC tissues was also demonstrated first in 2004.^63^ Later, in 2018, *AKR1B1* expression was reported to increase in HCC gradually from 4‐month nodules to 17‐month tumours in rat models of HCC.^64^ The elevated *AKR1B1* expression in HCC was also accompanied by other observations. Ectopic expression of *AKR1B1* in the Hep2G cell line has been demonstrated to increase cell proliferation, migration, invasion, colony formation and wound healing whereas suppression of *AKR1B1* caused the opposite effects.^65^ In contrast to these data, a study conducted in 2015 reported that the expression of *AKR1B1* in primary HCC tissues diminished in comparison with non‐tumour tissues as its promoter was heavily methylated.^66^

Overexpression of *AKR1B1* has been indicated to trigger the AKT/mTOR signalling pathway through interaction with the AKT1 kinase domain. It increased ‘Warburg effects, lactate production, oxidative stress and inflammation’ resulting in tumorigenicity in HepG2 cells. In the same study, the reduction of AKR1B1 led to a decrease in AKT/mTOR signalling and cancer development in mice. They suggested that due to the increased activity of the polyol pathway, more fructose might have been generated and converted to lactate, increasing the lactate levels in the cells. It is also possible that the AKR1B1 induced activation of AKT/mTOR signalling which ultimately resulted in increased flux of lactate created by aerobic glycolysis.^65^

Furthermore, it has been explained that triiodothyronine (T3) could promote *AKR1B1* expression in HepG2 cells which its amount in vivo is associated with the expression level of thyroid hormone receptor (TR) proteins. Additionally, the study has indicated that the T3 induction of *AKR1B1* expression did not rely on de novo protein synthesis. The levels of *AKR1B1* in cells induced by T3 did not show any noteworthy difference in the presence of a protein synthesis inhibitor such as Cycloheximide.^10^ *AKR1B1* promoter region has also been the subject of several studies in HCC. Mutations in the −1079/−1068 region of the *AKR1B1* promoter could abrogate the activation of this gene by T3. An atypical palindrome‐like TRE sequence has been identified. These data suggested that T3 induced *AKR1B1* expression is regulated by TR/TRE.^10^ Additionally, two antioxidant response element (ARE) sites in *AKR1B1*, *AKR1B10* and *AKR7A3* promoters have been found which could bind with NRF2, a TF that is involved in cellular defence against oxidative stress.^64^, ^67^ So, AKR1B1 could be involved in oxidative responses. Besides, induction of *NRF2* could silence human monocytic leukaemia cell line U937 and cause an increase in *AKR1B1* expression suggesting that *NRF2* may regulate *AKR1B1* expression in peripheral blood cells.^67^

#### 2.3.2 Down‐regulation of *AKR1B1* gene

##### Endometrial cancer

*AKR1B1* expression in endometrial cancer has been reported to be decreased and correlated negatively with body mass index (BMI) suggesting a decreased PGF2a formation.^23^, ^68^ The down‐regulation of AKR1B1 has been reported to be more in post‐menopausal samples in comparison to pre‐menopausal samples. Besides, AKR1B1 was localized in the cytoplasm of epithelial cells, which is in concordance with other reports about the epithelial cell‐specific expression of this gene. It has been proposed that adipose tissue could regulate the synthesis of pro‐inflammatory PGF2a via AKR1B1 regulation. Thus, AKR1B1 could be involved in the initiation of endometrial cancer through modulating inflammation.^23^, ^68^

##### Adrenocortical carcinomas

Excessive amounts of AKR1B1 has been seen in normal human adrenal tissue.^14^ However, *AKR1B1* expression has been demonstrated to be reduced in adrenocortical carcinoma (ACC), being less than adrenocortical adenomas and Cushing's hyperplasia. It has been proposed that cyclic adenosine monophosphate (CAMP) could regulate the expression of *AKR1B1* in adrenocortical cells. Forskolin, a CAMP synthesis activator, could increase *AKR1B1* expression.^14^ The tissue factor cAMP‐responsive element‐binding protein (CREB), adrenocorticotropic hormone (ACTH) and protein kinase A (PKA) activity induced by cAMP had decreased in ACC.^69^, ^70^, ^71^ The mechanism underlying AKR1B1 pathogenesis in ACC has not been established yet. However, inhibition of aldose reductase has been reported to cause elevated levels of HNE which could increase phosphorylation of CREB and cell proliferation.^71^, ^72^, ^73^ One hypothesis might be that HNE could form adducts in DNA, proteins or lipids of the body, important in cancer induction.^74^ Further research is needed to unveil its accurate mechanism in ACC.

## 3 FUTURE PERSPECTIVES

DNA methylation has been presented as a diagnostic biomarker for cancer detection with the advent of FDA approved tests such as Epi proColon and Cologuard, which could screen methylation of *SEPT9*, *NDRG4* and *BMP3* in CRC.^75^, ^76^, ^77^, ^78^ To find diagnostic, prognostic and therapeutic biomarkers with a higher performance for cancer, *AKR1B1* has been chosen as a subject of study by several researchers. Although *AKR1B1* expression has been found to be associated with tumour size in CRC,^35^ more evidence is needed to support *AKR1B1* expression as a CRC biomarker. In addition to gene expression, hypermethylation especially in the gene promoter, has been widely suggested as a diagnostic biomarker.^79^, ^80^ For example, *AKR1B1* has been shown to be highly methylated in CpG islands of its promoter, involved in dysregulation mechanisms of prostaglandin‐endoperoxide synthase.^81^, ^82^ Hypermethylation of *AKR1B1* and its negative correlation with mRNA expression have been displayed by in silico studies.^29^, ^83^ Consistent with these data, in a study using public methylation dataset GSE48684, *AKR1B1* methylation indicated an area under the roc curve (AUC) of 0.84 between normal and CRC tissues as well as an AUC of 0.874 between normal, adenoma and CRC tissues. The same study has also analysed GSE68060 dataset in which the AUC of *AKR1B1* was reported to be 0.954 alongside a 98 per cent value for the beta‐adducin (*ADD2*) gene. This suggests that methylation of these two genes could be used as a biomarker for screening and diagnosis of CRC.^83^ On the contrary, *AKR1B1* has been reported not to be suitable as a diagnostic biomarker for detecting lymph node metastasis as no significant differential expression between control and Dukes stage c group samples has been detected.^36^ In addition to *AKR1B1*, *AKR1B10* has also been investigated as a potential prognostic biomarker for CRC. It has been suggested that lower *AKR1B1* and higher *AKR1B10* expression indicate a good prognosis for this cancer and vice versa.^29^

*AKR1B1* could also be served as a diagnostic biomarker for breast cancer. For example, *AKR1B1* promoter has been reported to be highly methylated in breast cancer tissues.^84^, ^85^, ^86^ Besides, a study has demonstrated that *AKR1B1* methylation occurred specifically in epithelial breast cell lines.^85^ Another study has indicated that promoter hypermethylation of *AKR1B1* and *TM6SF1* could be used to detect breast cancer with an AUC of 0.986.^86^ Furthermore, although it is proposed that the methylation rate in nipple fluid is less than tumour tissues, researchers have been able to differentiate cancerous nipple fluid samples from healthy ones by analysing methylation of a gene panel including *AKR1B1*, *ALX1*, *RASSF1A* and *TM6SF1*.^87^ The limitation of this study was the selection of cases with different age groups in tumour and controls samples although there are no clear reports correlating of methylation and age in breast cancer.^86^, ^87^ Additionally, hypermethylation of *AKR1B1* has been observed in independent her2+ breast tumours in comparison with normal breast tissues.^84^ In ductal and lobular breast cancer, however, no correlation between cancerous and normal tissues in Oncomic analysis has been observed.^86^

This is also some evidence suggesting *AKR1B1* as a putative biomarker for hepatoma.^88^ Besides, there is a negative correlation between the ratio of tumoural *AKR1B1* expression to its normal tissue expression and liver cirrhosis.^88^

Altogether, these data suggest that *AKR1B1* methylation has the potential to be used as a diagnostic biomarker in breast cancer and CRC although further research with higher sample sizes is needed to provide more valid data.

## 4 BIOMARKER FOR PREDICTION

Anti‐cancer drug resistance is still one of the major concerns in the treatment of cancer. Drug resistance occurs in two ways; either poor initial response is seen because of the intrinsic resistance before exposing cells to drugs or through a good initial response followed by a poor outcome in which cells have acquired resistance against the drug later in the process of the treatment. The intrinsic drug resistance has been suggested to be more related to the alterations in drug breakdown, interactions of the drug with its target, transportation of the drug through the cell membrane, function of the drug in cells and efflux of the drug. In contrast, acquired drug resistance has been related to genetic and environmental factors which could lead to alterations in metabolic pathways or help resistant tumour cells to grow.^89^, ^90^ Several lines of information indicated that both up and down‐regulation of *AKR1B1* could take part in drug resistance in cancer. For example, in 1997, a group of scientists demonstrated that sorbitol elevation in cells could confer resistance to NSCLC cells against Cisplatin, suggesting that an increased activity of AKR1B1, which produces sorbitol, may have the same outcome.^91^ Additionally, it has been indicated that higher expression of *AKR1B1* could promote resistance to Daunorubicin (DRC) and Doxorubicin (DOX).^92^, ^93^ DOX is classified as an anthracycline antibiotic that could be used in the treatment of cancer.^92^, ^94^ In 2002, LEE *et al* demonstrated that inhibition of aldose reductase caused more sensitivity of HeLa cervical carcinoma cells to anti‐cancer drugs such as DOX and Cisplatin. The study suggested that the induction of ERK followed by AKR1B1 inhibition promoted the cells to become more sensitive to the drugs.^95^ However, researchers later highlighted that ATP‐driven effluxes, as well as carbonyl reduction, could be the main reasons for resistance to these drugs.^96^ Overexpression of multiple ATP‐binding cassette (ABC) transporters such as ABCG2, MDR1 and Multidrug Resistance Protein 1 (MRP1) has been shown in the resistant tumour cells.^97^ Using a combination of different drugs has been a common way to overcome the resistance caused by the efflux of the drug.^98^ For example, it has been demonstrated that a combination of tyrosine kinase inhibitors (TKIs) with chemotherapy drugs could help to overcome the drug resistance caused by overexpression of ABC transporters.^99^ In this regard, co‐delivery of DOX and Fidarestat has been shown to lower the MDR1, MRP1 and ABCG2 drug transporters, thereby reducing the drug efflux in tumour cells and decreasing the drug resistance.^100^ Furthermore, a prodrug nano assembly of Epalrestat and DOX has increased the uptake of DOX and synergically inhibited the cell growth and improved apoptosis.^101^

As a result of carbonyl reduction, less toxic compounds such as Doxorubicinol and Daunorubicinol are produced from DOX and DRC, respectively. This could provide cells with the ability to resist more to a cytotoxicity state.^92^, ^96^ For example, induction of AKR1B1 which could reduce DRC to Daunorubicinol induced resistant to tumour cells in pancreatic cancer.^93^ In addition, *AKR1B1* up‐regulation was suggested to protect the cells against DOX in MCF‐7 cells by converting this drug to Doxorubicinol, a less cytotoxic compound.^96^ Recently, it has been found that cyclin‐dependent kinase inhibitors such as Dinaciclib, Roscovitine, Purvalanol A, AZD5438 and R547 could inhibit AKR1C1 and to some extent AKR1B10, therefore sensitizing resistant cells.^102^, ^103^, ^104^ For example, Dinaciclib could synergize with DRC thus help overcome resistance to DRC in high AKR1C1 expressing cells by inhibiting AKR1C1 and lowering DRC reduction.^102^ A similar trend for AKR1B1 might emerge. On the other hand, lower expression of *AKR1B1*, associated with 2‐Deoxyglucose (2DG) that is an anti‐cancer drug and a substrate for AKR1B1, causes more drug resistance in tumour cells. Thus, it was proposed that tumour cell lines with lower levels of AKR1B1 such as SW480 and HT29 were more resistant to 2DG than tumour cell lines with higher levels of AKR1B1 such as HepG2 and SKOV3.^31^ In addition, inhibiting AKR1B1 in high expressing cell lines resulted in less sensitivity to 2DG cytotoxicity. Moreover, there is more evidence supporting that lower AKR1B1 could induce resistant of tumour cells to 2DG. In this regard, treatment of tumour cells with Tolrestat and Fidarestat, which are aldose reductase inhibitors increased cell resistance to 2DG cytotoxicity. Besides, treating SW480 and HT29 cells with carbobenzoxy‐Leu‐Leu‐leucinal (MG132) and bortezomib, which could activate aldose reductase and up‐regulate NRF2 and Cox‐2, followed by administration of 2DG after 24 h, resulted in a higher sensitivity to 2DG cytotoxicity. These data suggest that the mechanism of cell resistance to 2DG differs from that of DOX and DRC. 2DG is suggested to affect tumour cells by diminishing GSH levels, promoting oxidative stress and destructing the tumour cells. It has been demonstrated that both Tolrestat and Fidarestat could restore the GSH levels in 2DG treated cells. This may be because of the ability of AKR1B1 in using the NADPH needed for the synthesis of GSH. Also, Zhang et al demonstrated the antitumour activity of glyceraldehyde and diacetyl which are alternative substrates for AKR1B1 in vivo and further showed that cells with higher AKR1B1 levels were more resistant to these two compounds. These data suggest that glyceraldehyde, diacetyl and 2DG could reduce the amount of GSH in *AKR1B1* overexpressing cells, increasing the level of cytotoxicity. A kind of glyceraldehyde and diacetyl substrates is DL‐Buthionine‐sulfoximine (BSO), a drug that could inhibit the synthesis of GSH. BSO could enhance the level of cytotoxicity in cells with elevated *AKR1B1* levels. In addition, N‐acetyl‐cysteine, a substance that could induce GSH production has shown the cells to become more resistant to glyceraldehyde and diacetyl. These data propose that 2DG glyceraldehyde and diacetyl could kill tumour cells by lowering the amount of GSH, however, *AKR1B1* depletion may provide more NADPH for the synthesis of GSH and this may promote cell resistance against these drugs.^17^ In another study including 39 cell lines and 64 anti‐cancer drugs, *AKR1B1* expression alteration induced the tumour cells to become more sensitive to 23 out of 64 drugs, suggesting that *AKR1B1* expression could be a putative marker for chemosensitivity prediction.^32^ Table 1 summarizes the list of AKR1B1‐related drugs and their effects in in vitro and in vivo studies.

**Table 1:** List of AKR1B1‐related drugs and their effects in vitro and in vivo experiments

Drug name	Experiment status	Description	Ref.
Epalrestat	in vivo	The only FDA approved AKR1B1 inhibitor The only AKR1B1 inhibitor approved in Japan Phase II clinical trial in China on triple‐negative breast cancer	89, 105, 106
Epalrestat	in vitro (MDA‐MB231 and SUM159)	restored E‐cadherin expression suppressed invasion and migration reduced PGF2a synthesis, the formation of tumour spheres and the frequency of colonies	51
	in vivo (female SCID mice)	decreased the size of tumours suppressed lung tumour metastasis	
Epalrestat	in vitro (MDA‐MB‐231 and 4T1)	targeted co‐delivery of Epalrestat and Doxorubicin via a redox‐sensitive pro drug increased apoptosis increased stoppage of the cell cycle in the G2/M phase	101
Fidarestat	in vivo (Apc^Min/+^ mice)	decreased the number of polyps induced by high fat diet (HFD) abrogated the HFD induced expression of PCNA, β‐catenin and phospho‐NF‐κB P65 decreased Cox‐2, iNOS decreased AKT activation and increase PKC B2	38
Fidarestat	in vitro (HT29)	decreased COX‐2, iNOS, XIAP, survivin, β‐catenin and NF‐κB	39
	in vivo (male C57BL/KsJ‐db/db mice treated with AOM)	inhibited PKC‐β2, AKT, COX‐2 and iNOS	
Fidarastat	in vitro ( HT29)	down‐regulated Bcl‐xL, Bcl‐2, survivin, XIAP and FLIP to up‐regulated pro‐apoptotic proteins such as BAX led to release of cytochrome c and activation of caspases‐3 increased death receptors DR5 and DR4, thus, increased TRAIL‐induced cytotoxicity and induced apoptosis regulated AKT/PI3K through activation of forkhead transcription factor FOXO3a	45
Fidarastat	in vitro & in vivo	increased the cells responsiveness to oxidative stress decreased mitochondrial DNA damage suppressed tumour cells increased Nrf2 (synergy with EGF) increased the Nrf‐2 DNA binding activity and decreased Keap‐1 expression enhanced the activity of Nrf2 stimulated by EGF in vitro & in vivo, thus, helped the cells adapt to oxidative stress enhanced the mitochondrial biogenesis under oxidative stress PCG‐1α, Nrf1 and TFAM were up‐regulated	47, 107
Fidarastat	in vitro	increased AMP‐protein kinase (AMPK) phosphorylation decreased the phosphorylation of mTOR in SW480 increased the expression of p53	47, 107
Fidarestat	in vitro (HT‐29 & SW480) and in vivo	increase the sensitivity to DOX and its accumulation decreased MDR1, MRP1 and ABCG2 inhibited DOX adverse effects could be used as adjuvant therapy to enhance DOX efficacy	100
Fidarestat	in vitro (HUVEC)	reduced endothelial cell death induced by DOX prevented the oxidative stress and ROS formed by DOX induction abrogated the effect of DOX on the induction of the expression of ICAM‐1 and VCAM‐1 as well as the adhesion of monocytes restored nitric oxide (NO)‐levels and eNOS expression decreased by DOX soothed the activation of inflammatory responses such as NFκB and cytokines in HUVECs and in vivo prevented the cardiac hypertrophy and expression of eNOS, iNOS and 3‐Nitrotyrosine in tissues of the aorta averted cytotoxicity created by DOX in non‐cancerous tissues	108
Fidarestat	in vitro (HUVEC) & in vivo (Fischer 344 rats)	inhibited angiogenesis factors such as Ki67 inhibited invasion and migration induced by VEGF‐ and FGF hindered MMP2 and MMP9 as well as ICAM, VCAM prevented the secretion of ICAM, VCAM, MMP2, MMP9 and IL‐6 induced by VEGF‐ and FGF into culture media increased IFN‐γ reduced proliferation prevented pi3k activation, phosphorylation of AKT, activation of NFκB and protein‐HNE adducts induced by VEGF hindered migration, invasion and creation of cells into structures like capillary in rats led to decreased expression of CD31 and vWF	109
Gedunin (compound)	SCC131 (Oral Cancer) and Eahy926)	inhibited *AKR1B1* expression, ROS formation and hypoxia‐induced cell migration inactivated Akt, ERK and NFκB better anti‐cancer effects alongside Epalrestat treatment	110
Gedunin	in vivo (Syrian hamsters)	inactivated Akt and inhibitory kappa B kinase (IKK) inhibited PI3K/Akt and NF‐κB pathways suppressed hamster buccal pouch (HBP) carcinomas progression inhibited mir‐21 vascular endothelial growth factor and hypoxia inducible factor‐1 alpha (HIF‐1α)	111
Aglycone extract of Genistein	in vitro MDA‐231	down‐regulated AKR1B1	112
Extract of artichoke leaves (bracts)	in vitro (human monocytic leukaemia cell line THP‐1)	inhibited AKR1B1 and NFκB activity in human leukaemic monocytes diminished the expression of COX‐2 and MMP‐2	113
Vincristine and 5‐aza‐dC	in vitro	could not affect *AKR1B1* methylation but induced its expression in CRC	81
UPA (Ulipristal acetate)	In vitro	induced *AKR1B1* expression slightly in myometrial cells decreased *AKR1B1* expression in leiomyoma cells	114

## 5 CONCLUSION

Although pre‐clinical investigations on the role of AKR1B1 in cancer and the application of its inhibitors have shown promising results, the lack of clinical studies on AKR1B1 inhibitors on cancer has hindered the use of these drugs. Thus, there is an urge to conduct more clinical studies on the application of AKR1B1 inhibitors as adjuvant therapy on different cancers.

## CONFLICT OF INTERESTS

The authors declare no conflict of interest with respect to this research.

## AUTHOR CONTRIBUTION

**Reza Khayami:** Conceptualization (equal); Investigation (equal); Writing‐original draft (equal); Writing‐review & editing (equal). **Seyyed Reza Hashemi:** Conceptualization (equal); Investigation (equal); Writing‐original draft (equal); Writing‐review & editing (equal). **Mohammad Amin Kerachian:** Conceptualization (lead); Investigation (lead); Supervision (lead); Validation (lead); Writing‐original draft (lead); Writing‐review & editing (lead).

## CONSENT FOR PUBLICATION

All authors read and approved the final manuscript.

## Data Availability

The data that support the findings of the present study are available from the corresponding author upon reasonable request.
